# British Red Squirrels Remain the Only Known Wild Rodent Host for Leprosy Bacilli

**DOI:** 10.3389/fvets.2019.00008

**Published:** 2019-02-01

**Authors:** Anna-Katarina Schilling, Charlotte Avanzi, Rainer G. Ulrich, Philippe Busso, Benoit Pisanu, Nicola Ferrari, Claudia Romeo, Maria Vittoria Mazzamuto, Joyce McLuckie, Craig M. Shuttleworth, Jorge Del-Pozo, Peter W. W. Lurz, Wendy G. Escalante-Fuentes, Jorge Ocampo-Candiani, Lucio Vera-Cabrera, Karen Stevenson, Jean-Louis Chapuis, Anna L. Meredith, Stewart T. Cole

**Affiliations:** ^1^Royal (Dick) School of Veterinary Studies and Roslin Institute, University of Edinburgh, Edinburgh, United Kingdom; ^2^Moredun Research Institute, Edinburgh, United Kingdom; ^3^Global Health Institute, Federal Institute of Technology in Lausanne, Lausanne, Switzerland; ^4^Institute of Novel and Emerging Infectious Diseases, Friedrich-Loeffler-Institut, Federal Research Institute for Animal Health, Greifswald-Insel Riems, Germany; ^5^Département Homme et Environment, Centre d'Ecologie et des Sciences de la Conservation, Muséum National d'Histoire Naturelle, Paris, France; ^6^Agence Française pour la Biodiversité, Centre d'expertise et de Données sur la Nature, Muséum National d'Histoire Naturelle, Paris, France; ^7^Dipartimento di Medicina Veterinaria, Università degli Studi di Milano, Milan, Italy; ^8^Dipartimento di Scienze Teoriche ed Applicate, Università degli Studi dell'Insubria, Varese, Italy; ^9^School of Natural Sciences, Bangor University, Bangor, United Kingdom; ^10^Laboratorio Interdisciplinario de Investigación Dermatológica, Servicio de Dermatología, Hospital Universitario, Universidad Autonoma de Nuevo León, Monterrey, Mexico; ^11^Melbourne Veterinary School, Faculty of Veterinary and Agricultural Sciences, University of Melbourne, Melbourne, VIC, Australia; ^12^Institut Pasteur de Paris, Paris, France

**Keywords:** leprosy, squirrels, white-throated woodrats, PCR, *Mycobacterium leprae*, *Mycobacterium lepromatosis*

## Abstract

Eurasian red squirrels *(Sciurus vulgaris)* in the British Isles are the most recently discovered animal reservoir for the leprosy bacteria *Mycobacterium leprae* and *Mycobacterium lepromatosis*. Initial data suggest that prevalence of leprosy infection is variable and often low in different squirrel populations. Nothing is known about the presence of leprosy bacilli in other wild squirrel species despite two others (Siberian chipmunk [*Tamias sibiricus*], and Thirteen-lined ground squirrel [*Ictidomys tridecemlineatus*]) having been reported to be susceptible to experimental infection with *M. leprae*. Rats, a food-source in some countries where human leprosy occurs, have been suggested as potential reservoirs for leprosy bacilli, but no evidence supporting this hypothesis is currently available. We screened 301 squirrel samples covering four species [96 Eurasian red squirrels, 67 Eastern gray squirrels (*Sciurus carolinensis*), 35 Siberian chipmunks, and 103 Pallas's squirrels (*Callosciurus erythraeus*)] from Europe and 72 Mexican white-throated woodrats (*Neotoma albigula*) for the presence of *M. leprae* and *M. lepromatosis* using validated PCR protocols. No DNA from leprosy bacilli was detected in any of the samples tested. Given our sample-size, the pathogen should have been detected if the prevalence and/or bacillary load in the populations investigated were similar to those found for British red squirrels.

## Introduction

Leprosy is one of the oldest known diseases in humans and can be caused by *Mycobacterium leprae* and *Mycobacterium lepromatosis* (1, 2). While treatable with antibiotics, >200,000 new human cases are still recorded every year. Transmission is thought to occur during close and frequent contact with infected people, but the exact mechanism remains unknown (3).

Humans were thought to be the only host species susceptible to leprosy bacilli until cases of naturally occurring infection with *M. leprae* were identified in the 1970s in nine-banded armadillos (*Dasypus novemcinctus*) in the southern United States of America and Mexico (4, 5). *M. leprae* has been reported repeatedly and in multiple locations in the Americas in nine-banded armadillos. To a lesser extent, leprosy has been reported in wild chimpanzees (*Pan troglodytes*) and sooty mangabey monkeys (*Cercocebus atys*) in Africa and several primate species have been infected experimentally with leprosy bacilli (6–10). In the Southeastern United States of America, identical strains of *M. leprae* were detected in nine-banded armadillos and humans (11) but the frequency or route of transmission cannot be determined from the data currently available. It has been suggested that prolonged close contact to infected animals and ingestion of their flesh and blood may be risk factors for human infection (12).

More recently, both leprosy bacilli were identified in seemingly healthy as well as diseased Eurasian red squirrels (*Sciurus vulgaris*) in the British Isles where autochthonous human cases have not occurred for centuries (13, 14). The *M. leprae* strain identified in the Eurasian red squirrel population on Brownsea Island was found to be closely related to strains from human leprosy cases in medieval England while the *M. lepromatosis* strains isolated from British squirrels diverged from Mexican human strains about 27,000 years ago (13).

Moreover, within the family Sciuridae two other species have been infected experimentally with leprosy bacilli; Siberian chipmunks (*Tamias sibiricus*) and Thirteen-lined ground squirrels (*Ictidomys tridecemlineatus*) (15, 16), but there are no publications about natural infection or disease in wild individuals from either species.

No natural leprosy infection of other rodent species within or outside the British Isles has been described (17). In 2015, during the genome sequencing of *M. lepromatosis, two* human cases of *M. lepromatosis* infection in central America were linked to the possible consumption of meat from *rata de campo* or “field rat” (18). Rodent meat, as well as armadillo meat, has been reported to be eaten by patients suffering from Hansen's disease in Mexico. However, no studies have been conducted yet to detect leprosy bacilli in wild rodents captured for consumption in this country.

It is possible that rodents may be infected with leprosy bacilli in locations other than those previously reported. Our research therefore aimed to assess the presence of *M. leprae* and *M. lepromatosis* in several rodent species in Mexico, continental Europe and the British Isles.

## Materials and Methods

### Study Population and Sample Types

**Figure 1:**
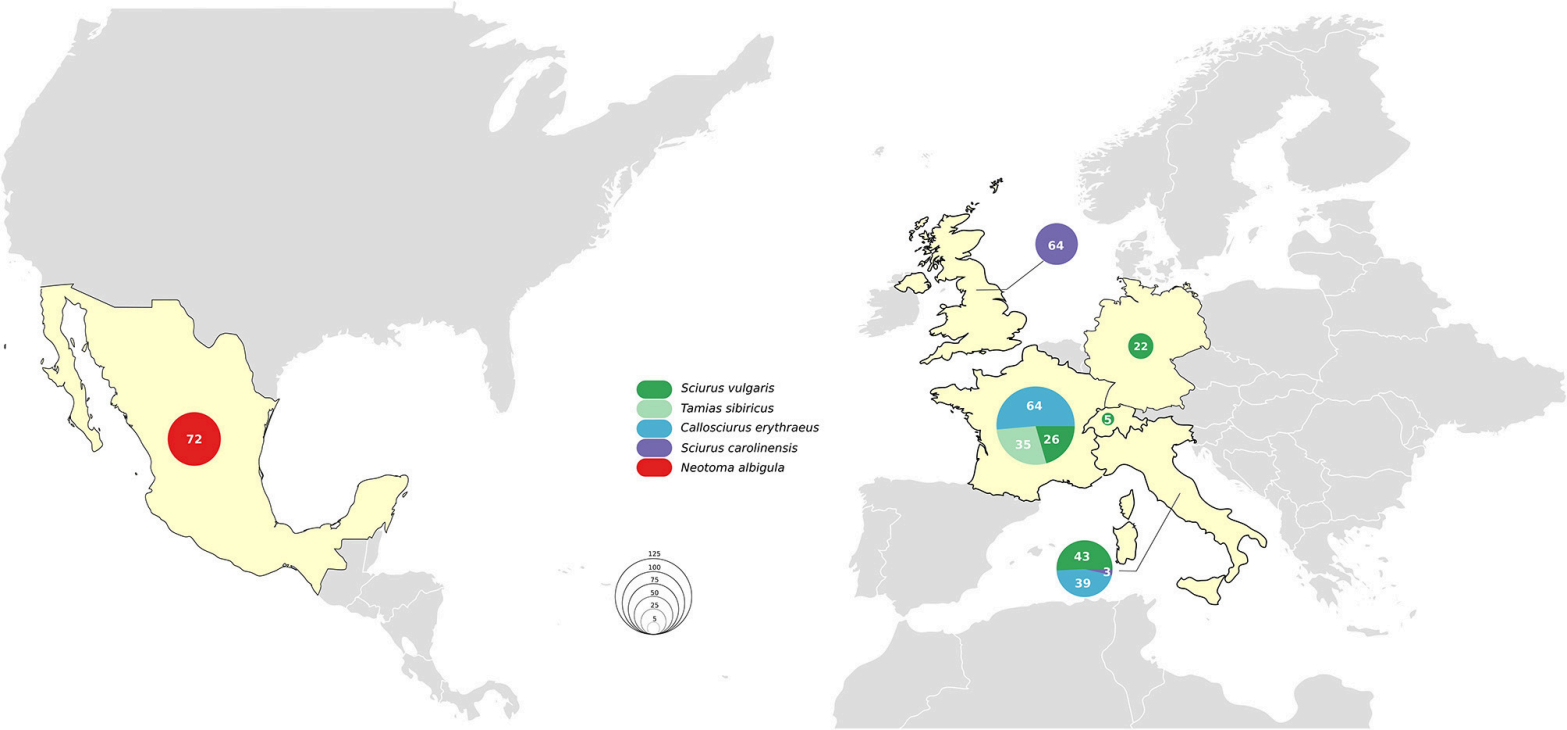
Countries of origin and species included in this study.

The six countries of origin and five different species included in this study are detailed in Figure 1. Animal carcasses were collected as part of surveillance efforts and were from animals that had been found dead, died at wildlife rescue centers, or had been humanely culled as part of invasive species control efforts. In Mexico, where human *M. lepromatosis* infections are reported (19), white-throated woodrats (*Neotoma albigula*) from the Matehuala region were bought at Monterrey meat market. Sample types included pinnae, footpads, liver, and lymph nodes (Table S1). When available, the pinnae samples were favored since it has been reported to be the most effective site for molecular screening (13). Samples were preserved in 70% ethanol for DNA extraction or in 10% formalin for histopathology.

### Histopathology

A 2–3 mm wide longitudinal section presenting the full length of the pinna was prepared. The trimmed tissues were submitted for Ziehl-Neelsen-staining according to the protocol described by Avanzi et al. (13). The entire sections were screened for acid-fast bacilli on a light microscope (magnification: 20× to 40×).

## DNA extraction and PCR Screening for the Presence Of Leprosy Bacilli

### Italian, German, British, and Mexican Samples

DNA was extracted using Qiagen DNeasy Blood and Tissue kit (Italian, German and British samples) or the Qiamp UCP Pathogen kit (Mexican samples) as previously described (13). PCR amplifications were performed in a 50 μl reaction volume containing GoTaq® G2 Flexi DNA Polymerase (1.25 Units/reaction, Promega) or Accustart PCR II SuperMix (Quanta Bioscience), 200 nM of primer pairs to amplify specific fragments of *M. lepromatosis* (LPM244) and *M. leprae* (RLEP 7 and 8) (13) and 3 μl of DNA extract. Sterile water was used as a negative PCR control and DNA extracts of *M. leprae* and *M. lepromatosis* from clinically diseased red squirrels as positive controls.

### Swiss and French Squirrel Samples

Total DNA was extracted from 10 to 100 mg of pinna tissue using the Extracta kit (Quanta Bioscience). PCR amplification was performed using the RLEP and LPM244 primer sets as described above. In addition, a primer set targeting the mitochondrial 12S ribosomal RNA gene of the Eurasian red squirrel genome was used to assess the performance of the Extracta DNA extraction method and to exclude the possibility of PCR inhibitors (13). For the other squirrel species, inhibitory effects were excluded by spiking pure *M. leprae* DNA (1 pg from strain Thai-53, BEI resources) into each sample followed by RLEP PCR.

### Statistics

Using WinPepi (v. 11.35; http://www.brixtonhealth.com/pepi4windows.html) DESCRIBE, we calculated the sample size necessary to find at least one case with 95% confidence if leprosy occurred at a prevalence of 21% in a population based on the estimated values currently available for clinically healthy squirrels in the British Isles (12). The necessary sample size based on this calculation is 19 individuals per population.

## Results

No *M. leprae* or *M. lepromatosis* DNA was detected in any samples from the 103 Pallas's squirrels, 96 Eurasian red squirrels, 67 Eastern gray squirrels, 35 Siberian chipmunks, and 72 white-throated woodrats analyzed by conventional PCR (Table 1, Figure 1, and Table S1). When Ziehl-Neelsen staining of selected tissues was performed, no acid-fast bacilli were observed (Table S1).

**Table 1:** Detailed origin, type and numbers of samples used in this study.

**Country**	**Area**	**Animal species**	**Body part**	**Number of samples (regional level)**	**Number of samples (national level)**
United Kingdom	Scottish borders	Eastern gray squirrel (*Sciurus carolinensis*)	Pinnae and footpads	48	64
	North Wales	Eastern gray squirrel (*Sciurus carolinensis*)	Pinnae and footpads	11	
	Purbeck	Eastern gray squirrel (*Sciurus carolinensis*)	Pinnae, footpads	5	
France	Sénart	Siberian chipmunk *(Tamias sibiricus)*	Pinnae	31	125
	Verneuil-sur-Seine	Siberian chipmunk *(Tamias sibiricus)*	Pinnae	4	
	Multiple departments	Eurasian red squirrel *(Sciurus vulgaris)*	Pinnae	26	
	Antibes	Pallas's squirrel *(Callosciurus erythraeus)*	Pinnae	32	
	Istres	Pallas's squirrel *(Callosciurus erythraeus)*	Pinnae	32	
Italy	Lombardy	Eurasian red squirrel *(Sciurus vulgaris)*	Pinnae	17	85
		Eastern gray squirrel *(Sciurus carolinensis)*		2	
		Pallas's squirrel *(Callosciurus erythraeus)*		39	
	Tuscany	Eurasian red squirrel*(Sciurus vulgaris)*	Pinnae	1	
	Piedmont	Eurasian red squirrel *(Sciurus vulgaris)*	Pinnae	25	
		Eastern gray squirrel *(Sciurus carolinensis)*		1	
Germany	Hesse	Eurasian red squirrel *(Sciurus vulgaris)*	Pinnae and footpads	19	22
	Hamburg	Eurasian red squirrel *(Sciurus vulgaris)*	Pinnae and footpads	1	
	Thuringia	Eurasian red squirrel *(Sciurus vulgaris)*	Pinnae and footpads	1	
	Bavaria	Eurasian red squirrel *(Sciurus vulgaris)*	Pinnae and footpads	1	
Switzerland	Vaud	Eurasian red squirrel *(Sciurus vulgaris)*	Pinnae	5	5
Mexico	Matehuala	*White-throated woodrat (Neotoma albigula)*	Liver, lymph nodes, muzzle, footpad	72	72

## Discussion

The recent discovery of Eurasian red squirrels infected with *M. leprae* or *M. lepromatosis* in the British Isles suggested that this rodent could be a reservoir throughout its range and that leprosy bacilli may have more hosts than was previously thought (13, 20–22). This is the first study to investigate the presence of leprosy bacilli in wild populations of squirrels outside the British Isles and in white-throated woodrats sold for consumption in markets in Mexico. It also expands the investigation into the presence of leprosy bacilli in Eastern gray squirrels in Great Britain. In this study, no animals representing five rodent species were found positive for leprosy bacilli DNA. It is therefore possible that only Eurasian red squirrels in the British Isles are a natural host for leprosy bacilli, which is consistent with the fact that to date no clinical signs of leprosy have been reported in wild populations of any squirrel species outside the British Isles. We calculated that a random sample size of 19 individuals should enable detection of at least one infected animal with 95% confidence in a continuous population where leprosy infection occurred at a rate of 21%, based on the estimated rate currently available for Eurasian red squirrels in the British Isles (13). Thus, our sample sizes should have been sufficient to detect at least one infected animal in the populations tested. However, we acknowledge that the prevalence of leprosy infection in Eurasian red squirrels varies widely depending on the geographical area in the British Isles (1–100%) suggesting possible local effects (13, 22). Our negative result could be due to the limited size of animal populations screened or to the limited geographical areas covered by our sampling. Testing larger numbers of squirrels throughout Europe would be necessary to state confidently that leprosy bacilli are absent. Additionally, while very specific tests for the detection of leprosy symptoms and bacilli exist, the sensitivity can be poor, especially for paucibacillary cases characterized by a low number of bacilli associated with few or no clinical lesions (23). Similar to humans, red squirrels display paucibacillary forms, which are difficult to detect even using the most sensitive PCR techniques (13). Serology is less reliable (13) and obtaining suitable blood samples from carcasses is problematic. PCR with tissue samples has so far been the most sensitive and specific method to identify leprosy bacilli in red squirrels (13, 20, 22). Nevertheless, only a small portion of tissue was screened for each animal with a preference for the pinnae, previously reported to be the most productive site for molecular screening (13). Investigation is ongoing to establish the best site and localization for effective detection of leprosy bacilli. Pending these investigations, false negative results cannot be excluded especially for paucibacillary cases. Moreover, our sample set included juvenile and sub-adult individuals, which may also limit the chance of detecting leprosy if disease progression is as slow as in the other known hosts (24). Currently, nothing is known about the timescale of leprosy bacilli infection and disease development in squirrels. It is, therefore, important to intensify research on leprosy progression in squirrels and to expand our detection toolkit to inform and enable future surveillance efforts.

Great Britain, Germany, Switzerland and France have been free of autochthonous human leprosy cases for decades or even centuries (14, 25–27), while autochthonous cases have been noted in more recent times in Italy (28). There are no obvious reasons why continental Eurasian red squirrels are less likely to have had contact with leprosy bacilli from a human source in the past than British squirrels, especially in countries where autochthonous human cases, albeit rare, still appear. For all non-indigenous squirrel species, the time of their introduction to Europe and the existence of leprosy in their native range may be informative. Gray squirrels, Pallas's squirrels, and Siberian chipmunks were introduced at different locations in Europe between 1870's and 1980's, long after human leprosy was eliminated from most European countries (29–32). When looking at their countries of origin, the Asian range of Pallas's squirrels does overlap with regions in which human leprosy is still endemic (33) and when the Siberian chipmunks were first introduced to Europe, human cases still existed in Korea for example (34). The natural range of Eastern gray squirrels does not overlap with areas where leprosy was endemic in humans. However, Eastern gray squirrels have replaced Eurasian red squirrels in large parts of the British Isles and currently share the same habitat in some regions where possible transmission or infection could occur as it has been shown for other pathogens (35, 36). However, we found no evidence of this. The white-throated woodrat samples came from an area in Mexico where autochthonous human leprosy cases occur and these animals are consumed as a food-source. Again, no evidence of infection was found.

Immunogenetics, which plays an important role in human and nine banded armadillo leprosy (37, 38), may also influence the susceptibility of squirrels to leprosy bacilli. Evidence exists for marked differences in disease resistance for different squirrel species (35, 39). Leprosy-specific evidence for this, in the form of polymorphisms in the key innate immunity toll-like receptor gene (*TLR1)*, was recently published (13). Indeed, certain *TLR1* polymorphisms were found more frequently in healthy red squirrels infected with leprosy bacilli compared to diseased animals implying that they may prevent the development of clinical disease. Further investigation of the functional genomics of different squirrel populations is required to understand why some Eurasian red squirrels are susceptible to leprosy bacilli while others are not, and whether higher genetic diversity on the European continent (39) prevents infection of Eurasian red squirrels here.

This is the first study to investigate the presence of leprosy bacilli in wild populations of squirrels outside the British Isles and in Mexican white-throated woodrats. The lack of detection of leprosy bacilli or clinical signs of leprosy was unexpected given the prevalence of leprosy in red squirrel populations in the British Isles. It shows that further targeted surveillance efforts and increased knowledge of the genetic determinants of leprosy resistance in animal hosts are necessary to fully understand the disease dynamics of leprosy in wildlife populations.

## Author Contributions

A-KS, CA, SC, J-LC, LV-C, AM, KS, PL, and JD-P conceived and designed the study. RU, J-LC, NF, CR, MM, BP, A-KS, WE-F, PL, CS, and JO-C collected samples. A-KS, CA, JM, PB, WE-F, and JO-C extracted DNA. A-KS, CA, JM, KS, PB, WE-F, and JO-C conducted molecular analysis. A-KS and JD-P conducted histopathological analysis. A-KS, CA, SC, KS, AM, PL, and JD-P analyzed and interpreted the data. A-KS, KS, CA, SC, and AM wrote the first draft of the manuscript. RU, CS, and PL wrote sections of the manuscript. All authors contributed to manuscript revision, read, and approved the submitted version.

### Conflict of Interest Statement

The authors declare that the research was conducted in the absence of any commercial or financial relationships that could be construed as a potential conflict of interest.

## Supplementary Material

Table S1 Overall dataset information and results.
